# Stakeholder recognition and response to human trafficking victims in Emergency Departments: a descriptive qualitative study in South Africa

**DOI:** 10.1016/j.afjem.2026.100983

**Published:** 2026-05-25

**Authors:** Leanne van Rooy, Tanya Heyns, Celia J Filmalter

**Affiliations:** Department of Nursing Science, School of Health Care Sciences, University of Pretoria, South Africa

**Keywords:** Human trafficking, Emergency department, Experiences, Person-centered trauma-informed approach

## Abstract

**Introduction:**

Human trafficking is currently an international crime and one of the most urgent human rights issues. Understanding stakeholders' experiences is critical to developing a care pathway that improves recognition and response. Globally limited qualitative research exists on stakeholders’ experiences of human trafficking in the Emergency Department. This study aims to explore and describe the experiences of stakeholders in recognizing and responding to victims of human trafficking.

**Method:**

Descriptive qualitative study reported using COREQ. Three online focus groups (29–40 min) were audio-recorded, transcribed, and thematically analyzed following Braun and Clarke.

**Results:**

The research team identified six codes: care pathways and policies for guidance, interprofessional collaboration, healthcare professionals’ responses, screening and triage, training and education, and a trauma-informed approach. These codes were then synthesized into three main themes: the need for clear guidance, including policies and procedural frameworks; education, covering screening, triage, recognition of red flags, and awareness; and interprofessional and multisectoral collaboration with coordinated referrals.

**Conclusion:**

Contextually appropriate, standardized care pathways for identifying and managing human trafficking victims are recommended for South African and broader African healthcare settings. These should be co-designed with healthcare professionals and survivors and aligned with existing systems to promote integrated care. Ongoing training and strengthened multisectoral collaboration are essential to ensure trauma-informed, coordinated, and effective responses.

## African relevance

• Contextually appropriate, standardized care pathways for identifying and managing human trafficking victims are recommended for African healthcare settings.
• Continuous awareness and ongoing targeted education using existing resources to ensure safe and effective care.
• Strengthening interprofessional multisector teamwork and institutional support is essential in low and middle-income countries to ensure coordinated, person-centered, and trauma-informed care.

## Introduction

Human trafficking is a form of modern-day slavery and violation of individual human rights [1,2]. Human trafficking is currently an international crime and one of the most urgent human rights issues [3]. The United Nations defined human trafficking in the Palermo Protocol of 2000 as follows:

*“The recruitment, transportation, transfer, harboring or receipt of persons, by means of threat or use of force or other forms of coercion, of abduction, of fraud, of deception, of the abuse of power or of a position of vulnerability or of the giving or receiving of payment or benefits to achieve the consent of a person having control over another person, for the purpose of exploitation.*” [4,1].

Worldwide, an estimated 50 million people live in modern slavery, with many remaining undetected and unsupported [5,6,1]. According to the Prevention and Combating of Trafficking in Persons (TIP) report, South Africa is a destination for human trafficking victims, including a source and transit point [7]. Johannesburg, Pretoria, Cape Town, Bloemfontein, and Durban are the five cities where syndicates currently operate [7].

Emergency Departments (ED) serve as access points for victims of trafficking, who may require medical care and present with urgent medical needs, injuries, psychological distress, or chronic health issues resulting from prolonged exploitation. According to the American College of Emergency Physicians in 2020, “*trafficking victims are treated for acute injuries and illnesses in EDs more often than in any other healthcare facility…*”. Approximately 50% to 87% of victims are never identified by healthcare professionals in EDs [8,1,9,10]. Healthcare professionals who recognize red flags should respond and treat them as possible victims [11,12,8,1].

Understanding stakeholders' experiences is critical for developing a care pathway to improve recognition and response to human trafficking [11,13]. Even though the healthcare sector plays a critical role in addressing human trafficking, qualitative research exploring stakeholder experiences in EDs is lacking. Globally and in low and middle-income countries, limited qualitative research exists on stakeholders’ experiences of human trafficking in the ED [14]. The researchers used a qualitative design to explore and describe the experiences of stakeholders in recognizing and responding to victims of human trafficking, aiming to identify barriers, enablers, and opportunities to enhance identification and response to this widespread public health and human rights issue.

## Methods

A descriptive qualitative design was used to explore stakeholders’ experiences of and responses to human trafficking victims presenting to Eds.

### Sampling

The researchers used maximum-variation purposive sampling to capture diverse perspectives among healthcare professionals working in EDs across different roles and sectors involved in acute care. Inclusion criteria were at least one year of ED experience, prior contact with suspected or confirmed human trafficking cases, and proficiency in English. Potential participants were invited to participate in online focus groups. The researcher then shared the participant information leaflet and consent form outlining the study's purpose. Three online focus groups were scheduled, and participants could choose the time that worked best for them. The COREQ checklist was used to ensure that the study conformed to the standards of qualitative research reporting.

### No patient or public contribution

This study included healthcare professionals who participated in online focus groups. Human trafficking survivors did not contribute to this article but are involved in the broader research study.

### Data collection

Online focus group interviews were conducted using Microsoft Teams. The researcher introduced herself, explained the study’s purpose, addressed questions, and reiterated the participants’ right to withdraw. The question asked was; *“What can be done to improve the recognition and response to human trafficking victims by healthcare professionals in the emergency department?”* (Supplementary data). Probing questions were not required during the focus group discussions, as participants in each group demonstrated high levels of engagement and were eager to share their insights, experiences, and recommendations. Their spontaneous contributions generated rich, in-depth data aligned with the core research question, thereby reducing the need for additional prompts. This active participation ensured a natural flow of dialogue and supported the co-construction of knowledge relevant to improving the recognition and response to human trafficking victims in the ED. The researcher did make field notes during the online focus groups. Informed consent was obtained from all the participants via email. Audio recordings were transcribed, with transcripts emailed to participants for validation. No amendments were requested. Counsellor was available to all participants during and after the focus group interviews.

### Data analysis

A qualitative thematic analysis was conducted using an inductive approach to explore stakeholders' experiences. The process followed the six-phase method outlined by Braun and Clarke [15]. The lead author transcribed all interviews verbatim and verified their accuracy by reviewing audio recordings. To deepen familiarity with the data and ensure comprehensive understanding, the transcripts were read multiple times. Coding was performed using ATLAS.ti. Version 25, incorporating all raw data to capture the full range of stakeholder input by the lead author. The authors reviewed the codes repeatedly, with insignificant ones removed to support a more nuanced interpretation of patterns and relationships. Research team developed three themes by identifying connections among the six codes and organizing them to reflect the underlying narratives within the dataset (Supplementary data). Each theme was then clearly defined and described in detail. The research team reached consensus with online meetings on the final thematic structure. The findings were then contextualized within the broader literature, forming the basis for discussion, recommendations, and conclusions.

### Rigor

Bias was minimized through collaborative analysis and reflective team discussions, ensuring a balanced interpretation of the data. Credibility was strengthened through member checking, where participants reviewed transcripts and confirmed the accuracy of interpretations [[16], [17], [18]]. Triangulation across diverse professional perspectives including counsellors, nurses, doctors, social workers, and a psychologist, provided a multidimensional understanding of the phenomenon. Reflexivity was maintained through documented reflections on the researcher’s positionality, including prior ED experience, direct exposure to human trafficking cases, and familiarity with institutional sexual assault policies, which informed but did not bias the analytic process. Dependability and confirmability were supported through detailed documentation of context, methodological decisions, and analytic procedures, creating a transparent audit trail using ATLAS.ti memos and a decision log. Data saturation was reached when no new insights emerged, participants consistently emphasized the need for a standardized care pathway, and highlighted significant gaps in education and training among healthcare professionals.

### Ethical considerations

Ethics approval was obtained from the University of Pretoria (711/2023), and the study was approved by the Institutional Research Committee (UNIV-2024-0015).

## Results

The study was a multi-site, multi-province qualitative study with ED clinicians from four Gauteng hospitals, plus key informants from Western Cape services and one non-governmental organization (NGO). Additional participants included an EMS provider from a university in the Western Cape, the Head of Clinical Forensic Medicine at a government hospital in the Western Cape, three doctors from private hospitals in Gauteng, and a psychologist from an NGO that assists survivors in Cape Town, with a total of 19 particpants. Due to the nature of the study, minimal demographics were collected to increase the confidentiality and possible re-identification of participants. Three online focus group interviews were conducted, lasting 38, 29, and 40 min each.

The research team initially identified six codes: (1) care pathways and policies for guidance, (2) interprofessional collaboration, (3) healthcare professionals’ responses, (4) screening and triage, (5) training and education, and (6) a trauma-informed approach. These codes were then synthesized into three main themes: (1) the need for clear guidance, including policies and procedural frameworks; (2) education, covering screening, triage, recognition of red flags, and awareness; and (3) interprofessional and multisectoral collaboration with coordinated referrals (Table 1).

**Table 1:** Themes and subthemes.

Theme	Sub-theme
1. Guidance	Policies to guide healthcare professionals
	Trauma-informed approach
	• Non-judgmental
	• Creating a safe environment
2. Education	Awareness of human trafficking
	Triage the 1st point of contact
	Red flags
	• Asking strategic questions
	• Observing nonverbal cues
	Screening tools and identification
	• Innovative and discreet strategies
3. Interprofessional multisector collaboration	Referral patterns
	• Handover from EMS

## Theme 1: guidance

Participants highlighted the urgent need for guidance and well-defined, standardized care pathways and protocols that specify appropriate actions, roles, and escalation procedures within clinical settings. As one counsellor mentioned, “*if there's some kind of protocol involved, it would change a lot of things*” (Counsellor 1), pointing out that the lack of structured procedures currently weakens effective responses.

Several participants further explained that, in practice, the lack of clarity regarding *“who the go-to people are”* (Doctor 4) leaves healthcare professionals feeling unprepared and unsupported when confronted with suspected trafficking cases. This uncertainty often results in inappropriate or unsafe management. As highlighted by a doctor, there is a clear need for structured ED policies that outline an organised, readily accessible system of response, ensuring that clinicians can act swiftly, secure the patient’s safety, initiate urgent referrals, and engage the appropriate support services before the perpetrator becomes aware of the intervention as supported by:

*“I think that would be very helpful to have such information to our EDs then there is an organized system that we can quickly access and we can quickly get a response… I think with such patient’s time would be of the essence before the perpetrator is actually aware of what's going on… Get the victim out of the ED, get the victim to safety.” (Doctor 2)*

The participants highlighted the importance of *trauma-informed care.* This involves creating non-judgmental, safe, and patient-centered environments that recognize the complex psychosocial needs of trafficking victims. Participants emphasized the importance of creating designated safe spaces in EDs and implementing flagging systems for at-risk patients. Nurses and counsellors mentioned the importance of slowing down the clinical pace, listening attentively, and building rapport to elicit disclosures that respect the victim’s autonomy and emotional state.

*“…creating a safe space, a non-pressurized space, a non-judgmental space, where you know patients can say that they might be in trouble and you know, sometimes it doesn't need to necessarily be in the department … how do you remove that person from that environment.”* (Doctor 2)

### Theme 2: education

Educating healthcare professionals will lead to increased awareness of potential victims. The first encounter is triage and identifying red flags, followed by screening tools to identify potential victims. Participants mentioned that healthcare professionals need a certain level of *awareness* to enable identification of potential victims in EDs. Healthcare professionals must be educated on recognizing red flags during triage and applying appropriate screening tools for possible victims.

*“ I see a fair number of intimate partner violence patients in our setting, but it has never popped that light bulb that this might be a human trafficking victim, so it is an eye-opener. It is something to consider myself to look forward to and try and just be more sensitive, or I wouldn't like to say suspicious, have that suspicion that or have that thought that this could be a human trafficking victim that I'm dealing with…”* (Doctor 3)

Education should include all healthcare professionals, from triage nurses to physicians, including administrative staff, to improve collective vigilance and information-sharing. Administrative staff, often the first point of contact, are key observers who may notice subtle signs and communicate concerns for further clinical assessment.

*“… if they're [administrative staff] also educated a bit about it, they can say, “You know, I don't know what that patient came in for, but these are the things that I saw. So, it's not that they are identifying that it's human trafficking, but maybe they've just picked up some key points…” (Counsellor 2)*

Participants mentioned that *triage* is an important entry point for identifying victims in EDs. Several participants observed that sharp triage nurses often raised suspicion and enabled early intervention, emphasizing the need to incorporate trafficking awareness into triage protocols and training.

*“… I just wanted to say that of the two cases that I knew, the one that was human trafficking was picked up by the triage nurse.” (Counsellor 1)*

*Screening and identification* tools are central to an effective response in the ED. Participants emphasized that the ED is a key contact point for trafficking victims, as stated by a participant: *“…up to 65% of victims interact with health services at some stage”* (Doctor 4). Innovative and discreet strategies were described, such as using coded markers (e.g., marking urine samples with a red pen) to safely flag patients in distress without alerting potential traffickers. Social workers emphasized the importance of asking strategic questions and observing nonverbal cues in identifying potential human trafficking victims.

*“…Patients will never say that I am a human trafficking victim because the perpetrators are usually the ones, the boyfriends, the people that are with them [victim] at the hospital at that time…”* (Social worker 1)

Participants suggested creating opportunities for patients to be seen privately, such as limiting family presence during initial consultation or triage, which was advocated as a practical safeguard to enable disclosure.

### Theme 3: interprofessional multisector collaboration

Participants highlighted the need for interprofessional and multisectoral collaboration to effectively identify, manage, and safeguard potential victims. Participants consistently noted that collaboration is vital within clinical teams and across emergency services, social welfare agencies, and legal entities to ensure appropriate and timely interventions.

*“…For me, it is training and it is networking…” (Doctor 4)*

Participants identified a lack of communication and accountability as significant barriers. This was evident in handover practices between EMS and healthcare professionals in the ED, where critical information about patients suspected of being trafficked should be communicated. “*The information either doesn't translate verbally or it's not well recorded on patient report forms,”* EMS 1 noted, adding that such breakdowns cause frustration and disillusionment. A lack of shared responsibility was apparent: "*We identified this person at risk in the ED… but nobody else wants to assist us"* (Doctor 3).

## Discussion

This study explored stakeholders' experiences in recognizing and responding to human trafficking victims who visited EDs. The findings reveal critical gaps in the South African healthcare system's ability to prepare, guide, and provide trauma-informed care, stressing the urgent need for interprofessional multisectoral collaboration and structured education programs for healthcare professionals in EDs.

**Fig. 1:**
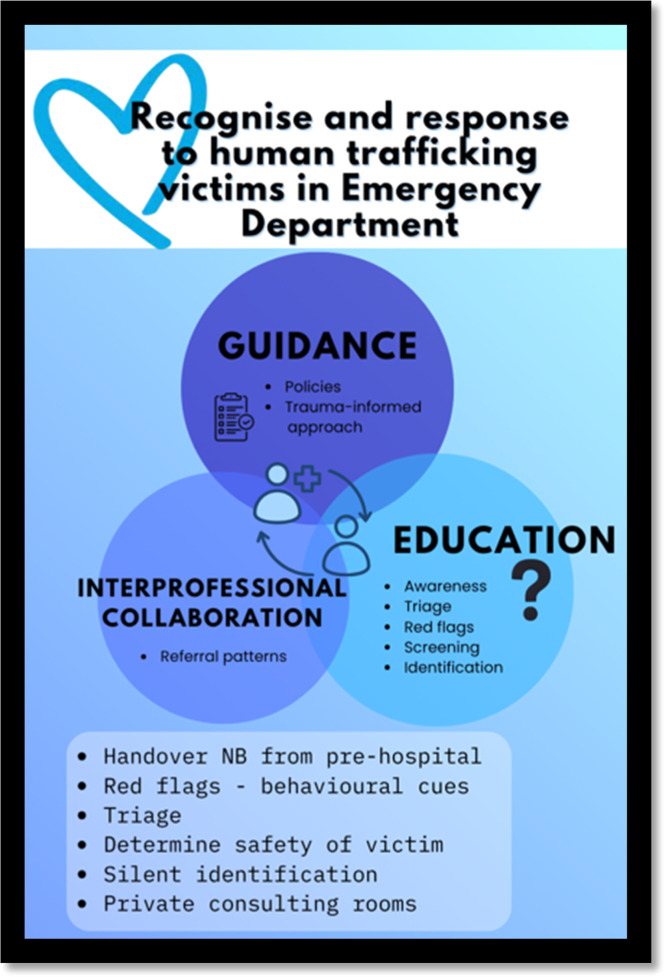
Conceptual framework.

A conceptual framework to support healthcare professionals is presented (Fig. 1). It emphasises three key components: guidance through policies and a trauma-informed approach, education on awareness, triage, red flags, screening, and identification, and interprofessional collaboration to ensure appropriate referral pathways. The framework outlines practical steps such as effective handover from pre-hospital services, identifying behavioural cues, ensuring patient safety, and creating opportunities for private assessment, reinforcing a structured and coordinated response to vulnerable patients.

### Guidance

Participants consistently reported a lack of formalized protocols, resulting in uncertainty about how to proceed once a potential victim is identified. These findings align with previous studies showing that the lack of institutional frameworks contributes to inconsistent, delayed, or unsafe responses in EDs worldwide and in Africa [5,19,20]. Traffickers may accompany their victims to EDs, which complicates safety and confidentiality, emphasizing the need for clearly defined standard operating procedures and protected spaces for disclosure [4,[21], [22], [23]]. The findings support the need to develop a standardized care pathway that should embrace a person-centered, trauma-informed approach, with continuous evaluation of effectiveness [19,24]. In the African context, where resources and training may be limited and trafficking routes are diverse, implementing such standardized pathways is especially important to provide timely, culturally sensitive, and safe care for victims in EDs.

### Education

Most healthcare professionals are unaware of the signs of human trafficking, often relying on intuition or experience. This lack of awareness aligns with international research showing that most trafficking victims remain unidentified due to limited training among healthcare professionals [19,23]. Triage nurses must be able to identify potential victims because screening is a vital step in EDs [25]. The South African Triage Score (SATS) system is used in South Africa [26]. Currently, no screening tools or self-identification measures are incorporated into triage. This absence of trafficking-specific screening represents a significant gap that directly limits the effective application of a trauma-informed approach within the South African ED setting.

Participants acknowledged that healthcare professionals frequently lack the knowledge and skills to implement trauma-informed practices and establish safe and supportive environments for victims [21,23]. Safe spaces in EDs should aim to provide a physical, dedicated private room away from traffickers and a psychologically safe space that encourages disclosure [4].

### Interprofessional multisector collaboration

In our study, poor collaboration between ED doctors and nurses, EMS and social workers resulted in information silos and led to missed opportunities for coordinated care. Global recommendations for managing trafficking in healthcare settings suggest an interprofessional system-based approach [25,21]. Appropriate referral networks are recommended with shared responsibility frameworks. The Human Trafficking Hotline at 0800 222 777 helps victims and the public report suspected trafficking cases and is managed by A21 in South Africa. Overall, the roles of DSD (Department of Social Development) and A21 represent a synergistic partnership within South Africa’s broader anti-trafficking architecture [27]. While DSD provides government-led statutory protection, welfare services, and policy coordination, A21 contributes specialised services, community-based prevention, and transnational expertise. This coordinated approach strengthens victim-centred responses, improves prevention strategies, and enhances the national capacity to identify, protect, and support persons affected by trafficking [27].

Where interprofessional collaboration is often challenged by limited resources, understaffing, and fragmented referral systems, strengthening multisector teamwork and institutional support is essential to ensure coordinated, person-centered, and trauma-informed care for trafficking victims in African ED’s.

This study explores the experiences of professionals involved in caring for human trafficking victims in EDs, highlighting both challenges and facilitating factors. By including EMS personnel, counsellors, and other stakeholders, the research extends beyond the ED and underscores the importance of collaborative, interprofessional, and multisectoral approaches. Despite using maximum variation sampling, certain key groups, such as police, special forces, and the pre-hospitalization sector, were not represented, and only a small number of government healthcare professionals participated in focus groups. Nonetheless, data saturation was achieved.

## Conclusion

Implementation of contextually appropriate, standardized care pathways for identifying and managing human trafficking victims within South African and broader African healthcare settings are recommended. These pathways should be co-designed with healthcare professionals and survivors, aligning with existing national frameworks, referral systems, and community resources to strengthen integration rather than establish separate care pathways. Continuous training and awareness initiatives are essential to equip healthcare professionals with the skills needed to respond effectively, ensuring the safety of victims and providing trauma-informed care. Moreover, strengthening multisectoral collaboration among healthcare professionals, social services, NGOs, and law enforcement is essential for coordinated, resource-efficient responses and to prevent fragmented or siloed care approaches.

## CReDiT author statement

LvR: Conceptualization, Formal Analysis, Methodology, Validation, Writing – original draft. CF: Conceptualization, Methodology, Supervision, Validation, Writing – review and editing. All authors approved the final version to be published and agreed to be accountable for all aspects of the work.

## Dissemination of results

The results of the whole PhD will be disseminated by giving feedback to all the healthcare professionals, survivors, and stakeholders who participated in the data analysis and co-designing a care pathway. The results will further contribute to a PhD dissertation, which will be made available online as well as in the library at the University of Pretoria.

## Declaration of competing interest

The authors declare that they have no known competing financial interests or personal relationships that could have appeared to influence the work reported in this paper.

## Appendix Supplementary materials

Image, application 1

Image, application 2
