# Supplementary figures and images for: Stakeholder recognition and response to human trafficking victims in Emergency Departments: a descriptive qualitative study in South Africa
Source: Afr J Emerg Med. 2026 May 25;16(3):100983. doi: 10.1016/j.afjem.2026.100983 (PMC13227186; doi:10.1016/j.afjem.2026.100983)

**Annexure B: Word cloud and coding of themes**


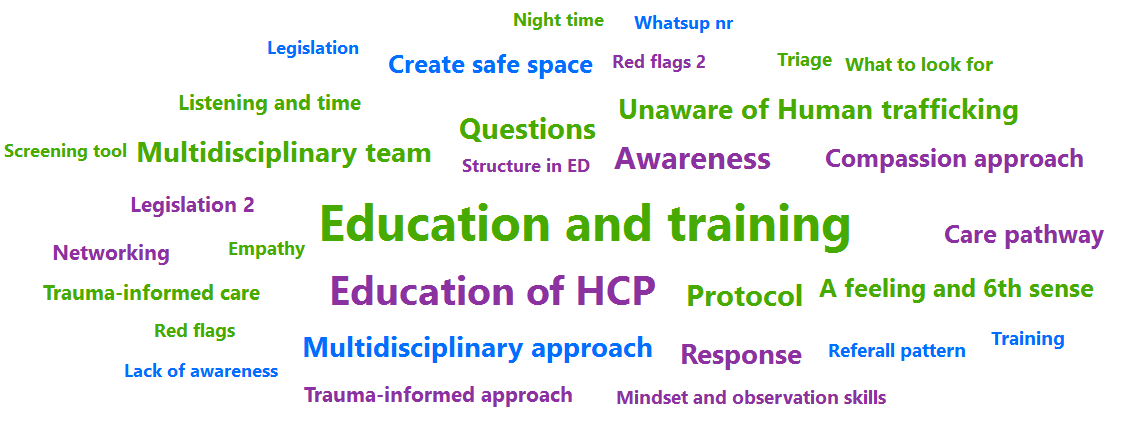


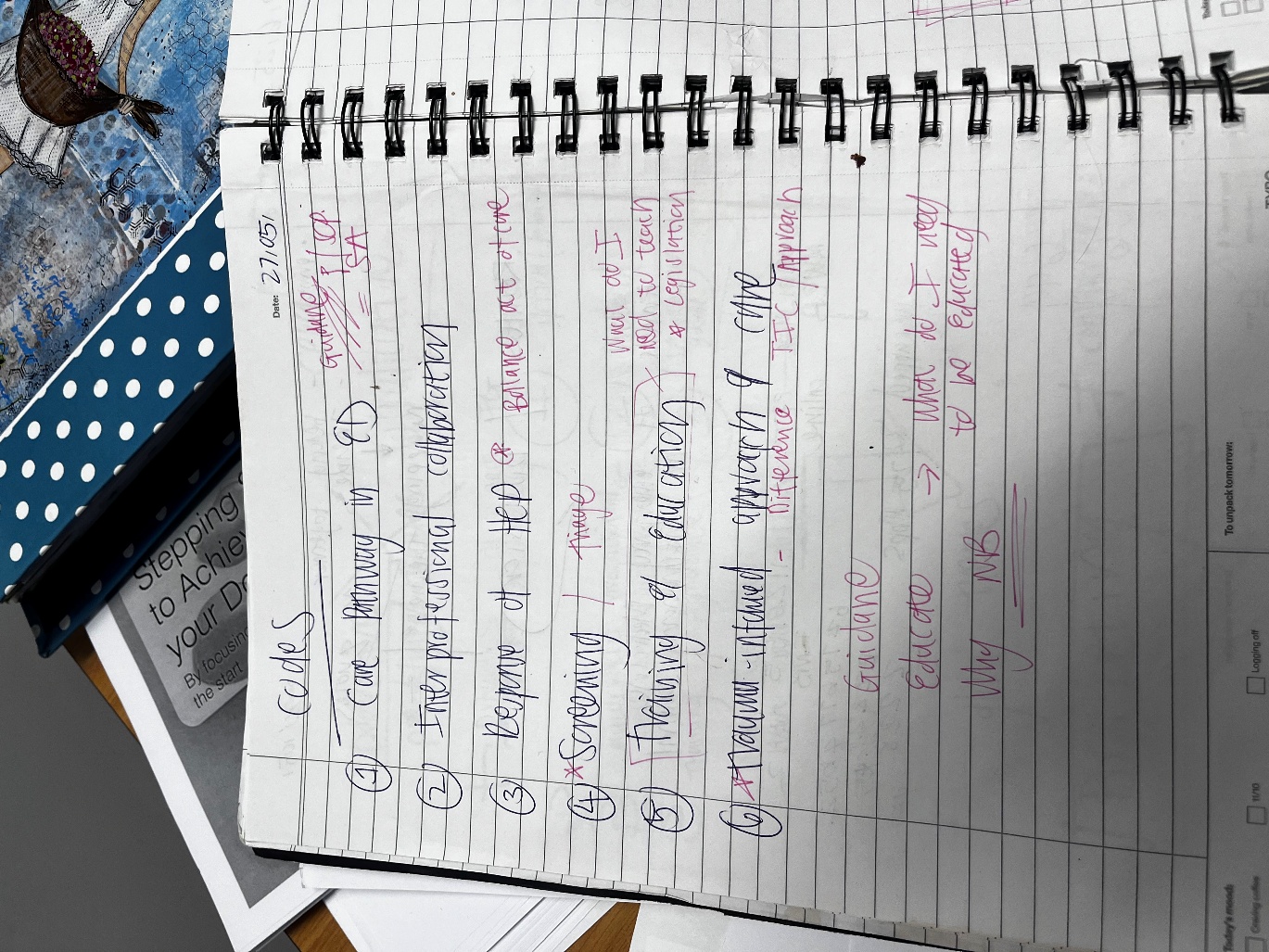


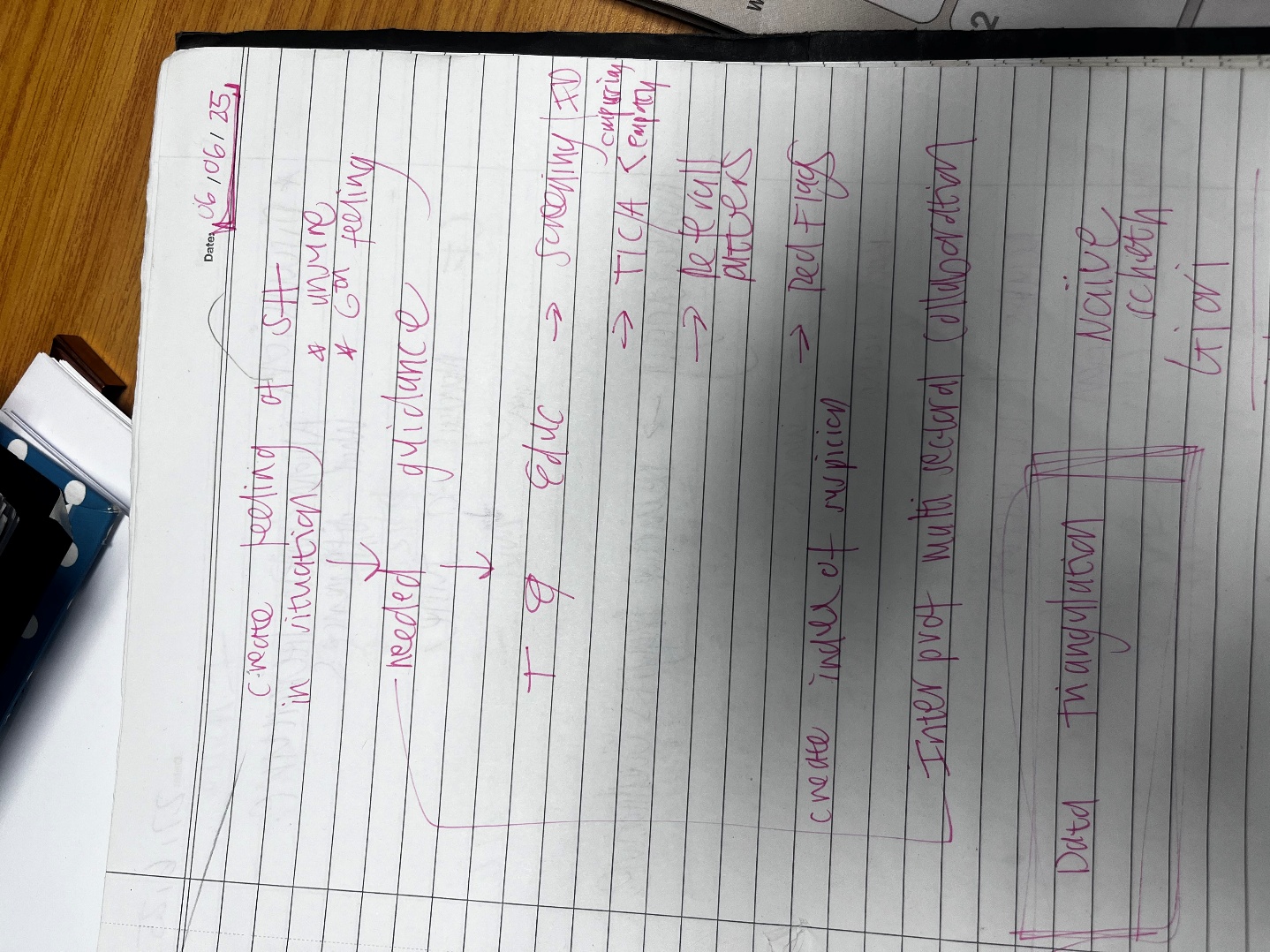

Supplement: Supplementary file 1 [file mmc1.docx]
