# Supplementary material for: Stakeholder recognition and response to human trafficking victims in Emergency Departments: a descriptive qualitative study in South Africa
Source: Afr J Emerg Med. 2026 May 25;16(3):100983. doi: 10.1016/j.afjem.2026.100983 (PMC13227186; doi:10.1016/j.afjem.2026.100983)
Supplement: Supplementary file 2 [file mmc2.docx]

**Annexure 1: Focus-group guide**

**Question**

What can be done to improve the recognition and response to human trafficking victims by healthcare professionals in the emergency department?

**Probing questions**

Can you provide an example of a situation where improved recognition strategies might have changed the outcome for a potential victim?

What specific tools or resources do you think would support healthcare professionals in responding more effectively?
